# Bone Health Status in Individuals with Amyotrophic Lateral Sclerosis: A Cross-Sectional Study on the Role of the Trabecular Bone Score and Its Implications in Neurorehabilitation

**DOI:** 10.3390/ijerph20042923

**Published:** 2023-02-07

**Authors:** Elisabetta Morini, Simona Portaro, Danilo Leonetti, Maria Cristina De Cola, Rosaria De Luca, Mirjam Bonanno, Angelo Quartarone, Rocco Salvatore Calabrò

**Affiliations:** 1 IRCCS Centro Neurolesi Bonino-Pulejo, S.S. 113 Via Palermo, C.da Casazza, 98124 Messina, Italy; 2 Physical and Rehabilitation Medicine Unit, Policlinico Universitario, 98125 Messina, Italy; 3 Department of Biomedical, Dental and Morphological and Functional Images, Section of Orthopaedic and Traumatology, University of Messina, 98122 Messina, Italy

**Keywords:** amyotrophic lateral sclerosis, osteoporosis, bone mineral density, trabecular bone score, osteopenia

## Abstract

*Background and Objectives*: Osteoporosis is a metabolic skeletal disease resulting in low bone mass with increased bone fragility and susceptibility to fractures. May lead to rapid loss of bone mineral density (BMD) due to physical inactivity and reduced muscle contractions. Generally, the diagnosis of osteoporosis is made using dual X-ray absorptiometry (DXA), by measuring BMD and the trabecular bone score (TBS), which can be useful for detecting bone fragility and susceptibility to fractures. Therefore, the aim of this study was to investigate, using BMD and TBS, the bone health status in a sample of amyotrophic lateral sclerosis (ALS) inpatients attending neurorehabilitation. *Materials and Methods:* Thirty-nine patients were included in the study and underwent electrocardiogram and blood tests, including calcium and parathyroid hormone, as well as vitamin D dosage, and DXA. *Results:* We found that the TBS of patients with osteoporosis was lower than that of those ALS patients with osteopenia or normal bone status, both in the lumbar spine and femoral neck, although no statistical significance was reached. In addition, Spearman’s correlation coefficient indicated a moderate correlation between TBS and lumbar spine BMD (r = −0.34) and a mild correlation between TBS and femoral neck BMD (r = −0.28). *Conclusions*: This study confirmed the hypothesis that ALS patients may exhibit deteriorated bone health with lower bone density and focused on the possible role of the TBS in the multidisciplinary approach to ALS.

## 1. Introduction

Osteoporosis is a metabolic skeletal disease resulting in low bone mass, reporting ≤ 2.5 standard deviations below peak bone mass, and abnormal bone structural architecture0 with increased bone fragility and susceptibility to fractures. Such osteoporotic fractures mainly occur in vertebrae, proximal femur and distal forearm and are responsible for significant reductions in patients’ quality of life, as well as increased disability, and social and economic burden [1]. The biological mechanisms of osteoporosis are most likely attributable to the augmented activity of osteoclasts and the reduced activity of osteoblasts, or both, determining an imbalance in bone health status, with rapid bone resorption and attenuated bone formation [2]. Further, osteoporosis can be classified as primary (including type I and type II) and secondary. Primary osteoporosis is common in post-menopausal women and in elderly people (both men and women over seventy years of age), whereas secondary osteoporosis is caused by systemic diseases, medical treatments or idiopathic diseases [3]. The menopausal transition represents a critical period for bone health in women. In fact, the decrease in bone mass is often accompanied by changes in the macro- and micro-structures of bone due to changes in sex steroid hormones, which increase bone resorption [4]. 

**Figure 1:**
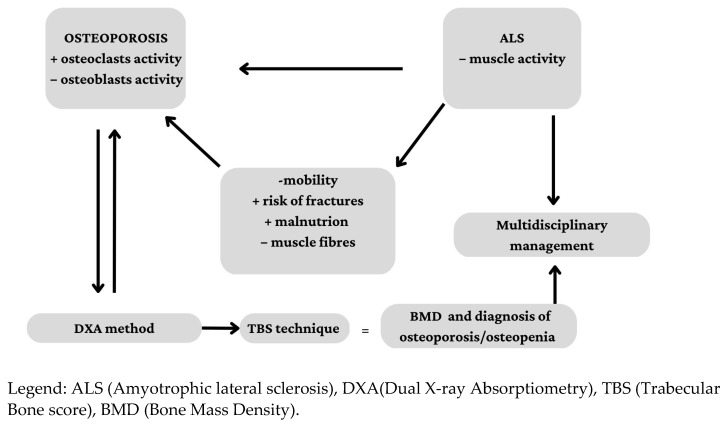
Interconnection among ALS, osteoporosis and its diagnostic procedures.

Neurological diseases may lead to rapid loss of bone mineral density (BMD) due to physical inactivity and reduced muscle contractions [5]. In fact, skeletal muscles provide certain anabolic stimuli for bone remodelling and are a source of osteogenic stem cells that play an important role in maintaining bone homeostasis [6]. Amyotrophic lateral sclerosis (ALS) is a neurodegenerative and still incurable condition characterised by severe muscle atrophy linked to the progressive loss of motor neurons. Clinically, ALS can be distinguished into two types, the bulbar and the medullary forms, depending on whether the upper or lower neurons are affected at onset, respectively [7]. In both ALS types, muscle weakness, disability, swallowing, breathing and walking alterations can occur, and often, respiratory failure is the final cause of death [8]. Musculoskeletal functional deterioration in ALS is associated with an increase in pathological bone fractures (i.e., induced by low-energy traumatic events) due to a decrease in bone density (Figure 1) [9,10,11]. 

Among other factors, ALS may also be associated with malnutrition and changes in energy expenditure, and this may lead to further muscle fibre loss as well as bone abnormalities [12]. Moreover, the increased risk of fracture may also be related to deficient intake of some minerals, such as calcium, and excessive intake of sodium and phosphorous [13]. Notably, the serum concentration of calcium is also influenced by Vitamin D (Vit D), which helps in calcium absorption in the bone. Vit D is a fat-soluble molecule that is introduced with the diet as well as through the skin and its exposure to sunlight; it is involved in maintaining muscle mass and strength [14], beside other important effects on the neurological system.

However, the aetiology of bone loss in ALS is under debate, even though several authors have hypothesised a multifactorial genesis (i.e., genetic causes, environment, reduced physical activity and frequent-fall risk/fractures) [15].

Generally, the diagnosis of osteoporosis is made using dual X-ray absorptiometry (DXA), which is the most widely used, non-invasive, quantitative diagnostic method for measuring bone density [16]. According to the International Society for Clinical Densitometry (ISCD), DXA is the recognized tool for skeletal health assessment, measuring BMD and bone mineral content (BMC) [17].

In detail, DXA allows a simple and safe examination to be performed using lower doses of dual X-rays than those of an X-ray or a CT scan; hence, it can be repeated safely over time. With DXA, clinicians can obtain information about BMD (expressed in g/m^2^) and images of total body, hip, posterior–anterior (PA) lumbar spine (LS) and/or forearm [18].

LS, total hip, femoral neck and/or 1/3 radius BMD values are usually considered for osteoporosis diagnosis and/or management. The decision to use each of them depends on the sex and age of the patient [19]. Based on the standard DXA method, the trabecular bone score (TBS) is an imaging technique that allows one to detect skeletal information additional to the standard BMD results. TBS analysis is performed as a computed evaluation of the pixel grey level in the lumbar spine derived from DXA images [20,21]. In this way, a low TBS indicates degraded bone micro-architecture, and studies have robustly shown that it can predict fractures independently of BMD and other clinical risk factors for fractures [22]. In this vein, the early assessment of bone health in ALS patients, i.e., since disease onset, could prevent osteoporosis-related complications thanks to the early start of anti-osteoporotic medication. In addition, the early diagnosis of osteoporosis allows special medication to be prescribed and a follow-up on nutritional recommendations or advice on how to improve bone mineral density to be offered. In fact, the bone health status plays a key role in multidisciplinary disease management, especially during rehabilitation sessions, where the goal of treatment, in ALS patients, is to prevent further muscle-skeletal damage while also enhancing patients’ life quality. However, currently, there is not enough evidence on the incidence/prevalence of osteoporosis/osteopenia in ALS patients.

Therefore, the aim of this study was to investigate the bone health status in a sample of ALS inpatients attending neurorehabilitation, using BMD and TBS, which are independent predictors of fragility/pathological fractures.

## 2. Materials and Methods

### 2.1. Study Population

**Figure 2:**
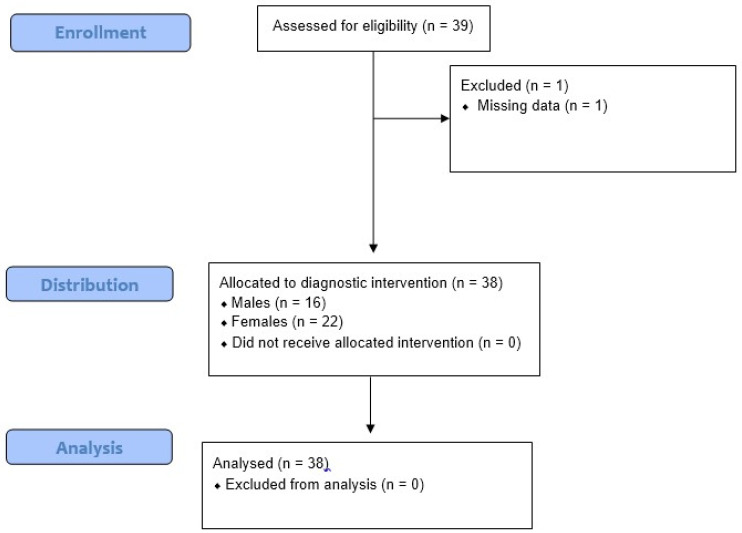
Flow chart for patient assessment in cross sectional studies, adapted from CONSORT diagram 2010.

Thirty-nine patients diagnosed with ALS according to the revised El Escorial criteria attending the Neurorobotic and Rare Disease Unit of IRCCS Centro Neurolesi “Bonino-Pulejo” (Messina, Italy) from September 2016 to February 2020 were enrolled in this study. One patient was excluded because of missing data (Figure 2).

Thus, the study population consisted of 38 individuals, 16 males (42%) and 22 females (58%), aged 42–83 years (61.82 ± 11.25).

To be included in the study, patients had to be able to walk unassisted and to be capable of giving written or verbal informed consent. Bedridden or severely affected patients and those with pulmonary and cardiac failure and/or with cognitive and behavioural abnormalities were excluded from the study. Individuals taking specific drugs, i.e., steroids, antiresorptive drugs, parathyroid hormone, thyroid hormone, calcitonin, oestrogen, glucocorticoids, thiazide diuretics, barbiturates, fluoride and antiepileptic drugs, were also excluded because of the potential interference with bone status. 

The study was approved by the Local Ethics Committee (IRCCSME 23/16), and the patients gave their informed consent to enter the study, after they were well informed about all the administered diagnostic procedures.

### 2.2. Procedures

After an accurate medical history (to investigate eating habits, previous vitamin deficiency or mineral metabolism alterations, hormonal abnormalities or sexual hormone supplementation, and familiarity for or previous bone fractures) and neurological examination, all patients underwent electrocardiogram and blood tests, including hemogram, kidney and liver enzymes, serum electrolytes (i.e., calcium, phosphorus, magnesium and sodium), and thyroid and parathyroid hormones, as well as Vitamin D concentration. The latter was measured using an automated electro-chemiluminescence assay (Roche).

**Figure 3:**
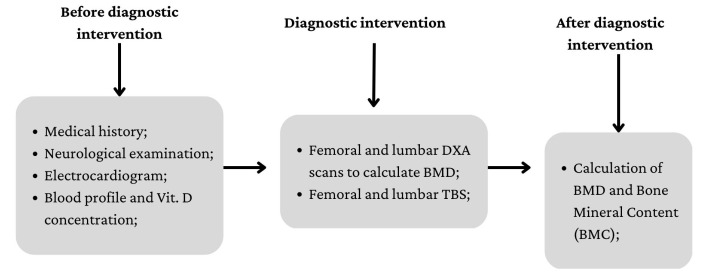
Data collection and diagnostic intervention process.

To classify patients in terms of osteopenia/osteoporosis, we performed BMD measurements using a DXA fan bean system (low X-ray emission) (Lunar Prodigy Primo—GE Medical Systems). Data collection and diagnostic procedure are summarized in Figure 3. 

The registered reports from densitometry measurements were expressed as T-score and Z-score, using the WHO criteria for DXA lumbar and femoral scans. Notably, according to the T-score values of the L1–L4 lumbar spine, patient BMD was delineated as normal (T-score ≥ −1 SD), low bone mass (osteopenia) (T-score between −1 and −2.5 SD) and osteoporosis (T-score ≤ −2.5 SD). 

On the other hand, the Z-score was used to exclude secondary osteoporosis based on the comparison of bone density with other people in the same age group and of the same size and gender. In fact, if the Z-score is excessively high or low, it may point out factors other than age that can affect BMD. 

We assessed BMD and BMC in the lumbar spine (LS) and femoral neck (FN) using a dual-energy X-ray absorptiometry (DXA) machine (Hologic–QDR 4500-W Discovery-A; Hologic Inc., Bedford, MA, USA). Based on the ISCD criteria [20], T-score ≤ −2.5 for the FN or LS defined osteoporosis; T-score between −2.5 and −1.0 identified osteopenia; T-score ≥ −1 indicated normal BMD. 

The trabecular bone score (TBS) was an additional evaluation performed while re-analysing the DXA LS (L1–L4) antero-posterior scans using Lunar Prodigy Primo—GE Medical Systems. The TBS was calculated as a medium value of the single-vertebra measurements (L1–L4) and their associations, excluding those affected by fractures, arthrosis and arthritis. 

### 2.3. Statistical Analysis

Analyses were performed using the 4.0.5 version of open-source software R(R Foundation for Statistical Computing.R Core Team; Vienna, Austria, 2022). Statistical significance was set at *p* < 0.05. Target variables (TBS, LS-BMD and FN-BMD) were examined for outliers using the Grubbs tests. Since the Kolmogorov–Smirnov test results showed normal distribution of the target variables, a nonparametric analysis was performed. Continuous variables were expressed as means ± standard deviations, whereas categorical variables were expressed as frequencies and percentages. The X^2^ test, the Mann–Whitney U test and the Wilcoxon rank sum test were used for comparison when appropriate. Correlations between quantitative variables were computed using Spearman’s coefficient.

## 3. Results

All patients completed the study but one (excluded for missing data) without side effects. They presented a nearly normal blood profile (but low vit D levels of 20 ± 9), and no significant gender differences emerged in either clinical scores or demographic variables, as shown in Table 1. 

According to the TBS values, patients were subdivided into three categories: NM-TBS (normal micro-architecture) when TBS ≥ 1350; PDM-TBS (partially degraded micro-architecture) when 1200 ≤ TBS < 1350; FDM-TBS (fully degraded micro-architecture) when TBS < 1200. The study sample included 13 (34.2%) patients with NM-TBS, 12 (31.6%) patients with PDM-TBS and 13 (34.2%) patients with FDM-TBS. Patients with FDM-TBS had lower mean age than those with NM-TBS or PDM-TBS, and patients in the LS-BMD and FN-BMD groups presented lower scores than the FDM-TBS group. However, no significant differences among these three groups were found (Table 2). 

However, we found that the femoral neck BMD values were significantly lower than lumbar spine BMD in all three classes (*p* < 0.01 for each comparison).

The BMD values (g/cm^2^) of the LS (L1–L4) were used to classify patients in the categories of normal (T-score > −1 SD), with osteopenia (−1 SD ≥ T-score > −2.5 SD) and with osteoporosis (T-score ≤ −2.5 SD). The comparisons between TBS and BMD are shown in Table 3 and Table 4, i.e., for lumbar spine and femoral neck, respectively.

Patients with osteoporosis had a lower TBS than those with osteopenia or normal patients, both in lumbar spine and femoral neck, although no statistical significance was reached. The mean age was quite similar in each group, but in the osteoporosis one, there were more females subjects over 50, which could have played a crucial role in the diagnostic procedures. Spearman’s correlation coefficient indicated a moderate correlation between TBS and LS-BMD (r = −0.34) and a mild correlation between TBS and FN-BMD (r = −0.28), i.e., the decrease in TBS was related to the increase in the BMD value. Moreover, a statistical correlation between 25-hydroxy vitamin D values and FN-BMD scores (r = 0.30) was also found.

## 4. Discussion

To the best of our knowledge, this is one of the few studies that evaluated the presence of bone fragility and susceptibility to fractures in ALS patients, using BMD and TBS methods. In fact, BMD is globally used as a valid technique to evaluate fracture risk; however, it is inexact in explaining why a person’s bones fracture and others’ do not. On the other hand, the TBS allows one to define individuals whose BMD is above the osteoporotic range as persons at higher risk of fractures [23]. More in detail, nearly normal or osteopenic bone (therefore, with a low risk of pathological fractures) may hide qualitative alterations in the trabecular structure (assessed only using the TBS), with a higher risk of fractures. Then, the TBS may be considered a complementary and useful tool to better assess such a risk profile.

In this cross-sectional study, we found that the poor bone health detected using the TBS could have been a consequence of age and changes in disease and could also have been related to the lifestyle (i.e., malnutrition, physical inactivity and lack of sun exposure) and to multisystem disease progression. In particular, we noticed that there was a correlation between lower TBSs and increased lumbar spine BMD, suggesting that trabecular bone thickness is associated with increased risk of fractures, despite the BMD results, as confirmed by other authors [24,25]. Moreover, low levels of physical activity or immobility can lead to cardiovascular deconditioning and muscle weakness, further contributing to reduced bone health status and strength of tendons and ligaments, which are also caused by the disease itself [26]. Notably, the muscular and bone systems in ALS patients could affect each other due to the lack of bone shape regulation induced by healthy muscles, which may alter bone morphology; such alteration, conversely, could worsen muscle degeneration and disease progression [15,27]. Among others, irisin seems to play a pivotal role, since it is secreted under the regulation of exercise and mediates the intercommunications between exercise and organs. In particular, it helps to reduce the body mass index by transforming white fat into brown and improves bone health by increasing the thickness of cortical bone [28]. As a limitation, we did not evaluate this important marker in our work. Future studies should investigate the role of irisin in ALS patients and also assess whether and to which extent its production (which should be reduced, given the muscle loss in the disease) may influence the bone health of this patient population. However, Lunetta, C., et al. [29] pointed out that circulating irisin in ALS patients is upregulated compared with healthy controls, suggesting a possible role of this myokine in the altered metabolic status due to the disease.

Moreover, our results revealed a positive correlation between Vitamin D and BMD. This link is almost under investigation, and there is no consensus on it. Some studies [30,31] have suggested that there is a direct relationship between low levels of serum vitamin D and low BMD and that it is associated with a worse prognosis. It is known that vitamin D, beside its fundamental role in favouring calcium absorption in bone, is involved in neuroprotection, in the modulation of neuroinflammation and in potentiating the effects of glia- and brain-derived neurotrophic growth factors [32]. In this scenario, ALS disease is related to nutritional issues due to feeding and swallowing problems, and reduced ingestion and intestinal absorption of the right vitamin D contribution, in addition to limited sun exposure, thus further contributing to increased risk of bone fragility and fractures [33,34]. However, Libonati et al. [35] showed that vitamin D supplementation did not have a significant effect on disease progression, explaining that this discrepancy is likely linked to inadequate physical activity due to ALS, and despite vitamin D supplementation, patients did not obtain those benefits observed in the physically active control group. 

With this in mind, how do we face BMD and BMC alterations in ALS? Do they play a functional role in prognosis? Considering that ALS clinical management implies a huge economic and social impact, although muscle impairment represents the main determinant, bone damage could play a role in functional prognosis and consequently in the level of quality of life [36]. To date, there is a growing body of evidence [37,38] indicating that the TBS is particularly useful as a complementary technique to BMD for fracture risk assessment in conditions associated with increased fracture risk. On such a basis, we propose to use the TBS in association with DXA scans to monitor bone health and to initiate early treatment with vitamin D supplementation in those who are deficient [39]. In fact, the TBS could be used retrospectively on DXA scans of patients to monitor bone status and to guide possible pharmacological approaches. 

Furthermore, this bone assessment could be particularly important in patients undergoing rehabilitation, where the risk of pathological fractures is higher because of the intensive training. In fact, growing evidence suggests that innovation technology, with regards to robotics, may potentiate functional outcomes in patients with neurological disorders [40]. In the last few years, attention has also been paid to the rehabilitation of neurodegenerative diseases, including ALS. Indeed, it has been suggested that a strictly monitored exercise program might significantly reduce motor deterioration in such patients, given that moderate-intensity exercise is beneficial to decreasing the deconditioning and muscle atrophy that can result from progressive inactivity. In this venue, the use of robotic devices, especially for gait impairment, is of potential benefit also in ALS individuals [41]. Promising results are also coming from studies on upper-limb robotic devices, as functional improvement has also been demonstrated [42]. Nonetheless, most of the robotic devices used to potentiate both upper- and lower-limb functions are exoskeletons, which exert high forces and friction on the joints with a higher risk of fractures, especially in patients with osteopenia/osteoporosis. Preventing and/or treating this bone pathology is, therefore, mandatory in all patients at risk of pathological fractures, including those with ALS, before training them with robotics [43]. Given that the TBS may detect those qualitative alterations that are not detectable by only using BMD, the tool should be used to better define pathological risk fractures in neurological patients receiving robotics-based assistance.

Finally, robotic training, especially if performed from the early stage of the disease, may balance the lack of physical activity in such patients, possibly even improving bone health [44]. Therefore, investigating the bone health status as part of the multidisciplinary rehabilitation approach is recommended, especially in the early stage of the disease, to start the right therapeutic choice for limiting BMD loss. To this end, future studies could compare more easy-to-use and non-invasive approaches, such as calcaneal quantitative ultrasound (QUS), to BMD/TBS in order to facilitate osteopenia/osteoporosis early diagnosis in the neurorehabilitation setting.

This study had some limitations to be acknowledged. First, the statistical significance of our results may have been influenced by the fact that significant changes in BMD may take time to manifest, which could represent a bias in our study, since ALS is a rapidly progressive condition. A prolonged follow-up should be recommended, consistent with the evolution of the disease. Moreover, the role of rehabilitation and its effects on bone health were not investigated, nor was the dosage of irisin levels, which could have given more strength to our data. 

## 5. Conclusions

In conclusion, this study confirmed the hypothesis that ALS patients may exhibit deteriorated bone health with lower bone density and focused on the possible role of the TBS in the multidisciplinary approach to ALS. Indeed, especially in the case of patients receiving robotic rehabilitation training, advances in techniques are required to achieve the early diagnosis of bone health deterioration and thus apply a multidisciplinary treatment strategy (pharmacological, clinical, nutritional, rehabilitative, etc.) with the aim of preventing fractures and improving patients’ quality of life.

## Figures and Tables

**Table 1:** Characteristics of the patients by gender. Data are expressed as means ± SDs or frequencies (percentages).

	Total (*n* = 38)	Male (*n* = 16)	Female (*n* = 22)	*p*-Value
Age (years)	61.82 ± 11.25	64.31 ± 12.91	60.00 ± 9.77	0.293
Over 50	31 (81.58)	14 (87.50)	17 (77.27)	0.705
Menopausal	19 (50.0)	-	19 (86.4)	-
TBS	1283.7 ± 151.3	1313.4 ± 176.6	1262.1 ± 130.0	0.487
LS-BMD	1.06 ± 0.21	1.09 ± 0.23	1.03 ± 0.19	0.544
FN-BMD	0.76 ± 0.18	0.80 ±0.18	0.73 ± 0.17	0.214

Legend: TBS = trabecular bone score; L-BMD = lumbar spine bone mineral density; FN-BMD = femoral neck bone mineral density.

**Table 2:** Characteristics of the patients by TBS category. Data are expressed as means ± SDs or frequencies (percentages).

	FDM-TBS (*n* = 13)	PDM-TBS (*n* = 12)	NM-TBS (*n* = 13)	*p*-Value
Age (years)	59.46 ± 11.63	65.17 ± 10.05	61.08 ± 12.01	0.307
Over 50	10 (76.92)	10 (83.33)	11 (84.61)	0.864
Female	8 (61.54)	8 (66.67)	6 (46.15)	0.553
LS-BMD	1.13 ± 0.22	1.03 ± 0.23	1.01 ± 0.82	0.241
FN-BMD	0.82 ± 0.21	0.74 ±0.16	0.71 ± 0.15	0.333

Legend: FDM-TBS = fully degraded micro-architecture; PDM-TBS = partially degraded micro-architecture; NM-TBS = normal micro-architecture; LS-BMD = lumbar spine bone mineral density; FN-BMD = femoral neck bone mineral density.

**Table 3:** Characteristics of the patients by LS-BMD classification. Data are expressed as means ± SDs or frequencies (percentages).

	Osteoporosis (*n* = 11)	Osteopenia (*n* = 11)	Normal (*n* = 16)	*p*-Value
Age (years)	62.54 ± 12.18	62.09 ± 13.49	61.12 ± 9.52	0.694
Over 50	9 (81.82)	8 (72.73)	14 (87.50)	0.623
Female	8 (72.73)	6 (54.54)	8 (50.00)	0.484
TBS	1270.3 ± 160.7	1298.5 ± 166.7	1282.7 ± 142.9	0.163

Legend: TBS = trabecular bone score; LS-BMD = lumbar spine bone mineral density.

**Table 4:** Characteristics of the patients by FN-BMD classification. Data are expressed as means ± SDs or frequencies (percentages).

	Osteoporosis (*n* = 18)	Osteopenia (*n* = 12)	Normal (*n* = 8)	*p*-Value
Age (years)	61.44 ± 11.73	61.67 ± 13.15	62.87 ± 7.83	0.536
Over 50	14 (77.78)	9 (75.00)	8 (100.00)	0.313
Female	10 (55.56)	7 (58.33)	5 (62.50)	0.946
TBS	1268.8 ± 151.3	1353.7 ± 171.7	1329.2 ± 107.8	0.242

Legend: TBS = trabecular bone score; FN-BMD = femoral neck bone mineral density. *p* < 0.05.

## Data Availability

Data will be available on demand from the corresponding author.
